# Licensed and Recommended Inactivated Oral CholeraVaccines: From Development to Innovative Deployment

**DOI:** 10.3390/tropicalmed6010032

**Published:** 2021-03-09

**Authors:** Jacqueline Deen, John D. Clemens

**Affiliations:** 1 Institute of Child Health and Human Development, National Institutes of Health, University of the Philippines, Pedro Gil Street, Ermita, Manila 1000, Philippines; jldeen@up.edu.ph; 2 International Centre for Diarrhoeal Disease Research, GPO Box 128, Dhaka 1000, Bangladesh; 3 UCLA Fielding School of Public Health, 650 Charles E Young Drive South, Los Angeles, CA 90095-1772, USA

**Keywords:** cholera, oral cholera vaccine, efficacy, effectiveness

## Abstract

Cholera is a disease of poverty and occurs where there is a lack of access to clean water and adequate sanitation. Since improved water supply and sanitation infrastructure cannot be implemented immediately in many high-risk areas, vaccination against cholera is an important additional tool for prevention and control. We describe the development of licensed and recommended inactivated oral cholera vaccines (OCVs), including the results of safety, efficacy and effectiveness studies and the creation of the global OCV stockpile. Over the years, the public health strategy for oral cholera vaccination has broadened—from purely pre-emptive use to reactive deployment to help control outbreaks. Limited supplies of OCV doses continues to be an important problem. We discuss various innovative dosing and delivery approaches that have been assessed and implemented and evidence of herd protection conferred by OCVs. We expect that the demand for OCVs will continue to increase in the coming years across many countries.

## 1. Introduction

Cholera remains a threat to many impoverished populations around the world. The long-term public health strategies against cholera and other enteric diseases are the establishment of safe water sources and the improvement of sanitation and hygiene (WASH). However, these measures are years away in many areas where cholera strikes, especially when war, political upheaval or natural disasters such as earthquakes and floods occur. The oral cholera vaccine (OCV) is an important complementary tool for cholera prevention and control. 

In this article we describe the history of the development of licensed oral cholera vaccines (OCVs). We discuss the accumulation of evidence on OCV safety, efficacy and effectiveness leading up to the recommendation by the World Health Organization (WHO) on mass oral cholera vaccination as both pre-emptive and reactive strategies. We discuss the initiation and expansion of the global OCV stockpile and its support by the Global Alliance for Vaccines and Immunizations (Gavi Alliance). We review various dose-sparing approaches (single-dose mass campaigns, targeting of specific high-risk groups and ring vaccination), evidence of herd protection conferred by OCVs and new delivery strategies. This article focuses on internationally licensed and recommended inactivated OCVs used during cholera outbreaks and in cholera-endemic sites.

## 2. Search Strategy

For this narrative review, we searched PubMed using the terms “oral cholera vaccine”, “cholera outbreak response” and “cholera vaccination campaign”, restricted to publications in English. We reviewed and included (a) relevant articles on the history of the development of inactivated OCVs, (b) publications during the last ten years on various innovative dosing and delivery strategies and (c) evidence of herd protection conferred by inactivated OCVs.

## 3. Development of Oral Cholera Vaccines and the Recommendation for Use

Injectable killed whole cell *Vibrio cholerae* O1 vaccines were widely available for many years [1]. These vaccines had poor efficacy and high reactogenicity and have not been recommended since the 1970s [2]. In the 1980s, a killed OCV consisting of inactivated whole cells of *V cholerae* O1 and the B-subunit of the cholera toxin (WC/rBS) was developed in Sweden [3]. Large scale trials of the vaccine in Bangladesh and Peru showed that the WC/rBS and the killed whole cell formulation alone were safe and conferred significant protection for up to 3 years [4,5]. An initial efficacy of 85–90% was obtained with the WC/rBS, declining to about 50% after 6 months. The oral vaccine without the B-subunit gave a somewhat lower initial level of protection but after 6 months the protection afforded by the two vaccines was similar. The WC/rBS vaccine is marketed as Dukoral (Valneva, Lyon, France) and is administered to those two years of age and older, as a two-dose regimen with a buffer (Table 1). Dukoral was the first OCV to obtain international licensure (in 1991) and WHO prequalification (in 2001). At that time, the WHO recommended inclusion of the WC/rBS vaccine among the tools to prevent cholera in populations believed to be at risk of cholera epidemic within 6 months and not experiencing a current outbreak [6]. 

The manufacturing technology of the Swedish vaccine was transferred to Vietnamese scientists at the National Institute of Hygiene and Epidemiology in Hanoi. A two-dose regimen of the first generation monovalent (anti-O1) OCV, containing only killed cholera whole cells and produced at USD 0.10 per dose in Vietnam, showed that it conferred 66% protection in a trial in Hue [7]. In 1997, killed *V. cholerae* O139 whole cells were added to the Vietnamese OCV due to the emergence of the new form of epidemic cholera caused by this serogroup. A bridging study found the bivalent (O1 and O139) OCV to be safe and immunogenic in adults and children one year and older [8]. The bivalent OCV was locally licensed as ORC-Vax (Vabiotech, Ha Noi, Viet Nam). The Vietnamese OCV has been used extensively in the Viet Nam public health system through mass immunization of high-risk populations. The burden of cholera in Vietnam has declined significantly in recent years, associated with widespread deployment of OCV and improvements in socioeconomic and WASH conditions [9]. The Vietnamese OCV has several distinct advantages over the original Swedish vaccine. Without a B-subunit component, the 2-dose Vietnamese OCV is easier and less expensive to manufacture, has less stringent cold chain requirements and is administered without a buffer.

The International Vaccine Institute (IVI) worked with VaBiotech to modify the strain composition of the bivalent OCV and improve the manufacturing process to conform with WHO standards [2]. The modified bivalent OCV was found to be safe and immunogenic in trials in Vietnam and India [10,11]. In 2009, the reformulated vaccine was licensed as mORC-Vax (Vabiotech, Viet Nam) but is not pre-qualified by WHO. To facilitate the international availability of mORC-Vax, manufacture of the reformulated vaccine was transferred to Shantha Biotechnics in India [12]. This led to the development of Shanchol (Shantha Biotechnics, Andhra Pradesh, India). A randomized, placebo-controlled trial in Kolkata, India showed that Shanchol is safe and confers 67% protective efficacy against cholera within two years of vaccination [12], 66% at three years [13] and 65% at five years [14] of follow-up. Shanchol, given as a 2-dose regimen to those one year of age and older, was licensed in India in 2009 and received WHO pre-qualification in 2011 (Table 1). 

By then, the majority of countries reporting cholera to the WHO were in Sub-Saharan Africa [15]. A large and protracted cholera outbreak spread all over Zimbabwe from 2008 to 2009 and resulted in 98,585 cases and more than 4000 deaths [16], as well as increasing pressure by the global public health community to deploy OCVs reactively [17,18]. With amassing evidence on OCV safety and efficacy and data on field effectiveness and feasibility of OCV mass vaccination in an African setting [19,20,21], in October 2009, the WHO Strategic Advisory Group of Experts (SAGE) on immunization recommended that oral cholera vaccination should be considered as a reactive strategy during outbreaks, in addition to the already recommended preventive use of OCV in endemic areas [22]. 

The recommendation on reactive use is very important since where and when a cholera outbreak will occur is difficult or impossible to predict. Reactive mass oral cholera vaccination was documented to be feasible and effective as an outbreak response in Guinea [23,24]. Following an initial hesitation to deploy OCV in Haiti shortly after the catastrophic 2010 earthquake [25], a large reactive mass oral cholera vaccination campaign in Haiti was shown to be successful despite logistic challenges [26,27]. An increasing number of reactive mass oral cholera vaccinations has been successfully conducted in different areas around the world under diverse circumstances [28].

With the broadening of the recommendation for oral cholera vaccination, the most important concern is ensuring a sufficient and sustainable supply of OCV doses. In September 2011, the WHO convened a meeting of experts at which an OCV stockpile was affirmed as necessary and feasible [29] and an OCV stockpile was created in 2012 [30], with pivotal support from Gavi starting in 2014 [31]. From 2013 to 2017, over 25 million doses were requested from the cholera vaccine stockpile, of which only 51% could be allocated and shipped to countries for 46 deployments [32]. Due to the limited number of OCV doses available, supplies were prioritized for cholera outbreaks, making preventive OCV campaigns difficult to plan and carry-out. To expand the global OCV production capacity, Euvichol (Eubiologics, Gangwon-do, South Korea), was developed based on the same formulation as Shanchol through a technology transfer from IVI. After a Phase I trial in Korea [33] and a bridging non-inferiority immunogenicity study in the Philippines [34], Euvichol was licensed and WHO-prequalified in December 2015 [35] (Table 1). Availability of Euvichol increases the number of affordable OCV doses that can be distributed through the stockpile to affected populations [35].

In 2018, Gavi’s Board approved an additional investment for pre-emptive OCV use in high-risk areas, which will become available in 2021, while continuing its support for OCV emergency use [31]. The current objectives of the GAVI investment include ongoing prevention of an OCV low demand–low supply cycle, reduction in cholera outbreaks in Gavi-supported countries and strengthening of the evidence base for periodic, pre-emptive campaigns [31]. Currently, the International Coordinating Group (comprising representatives from Médecins Sans Frontières, the International Federation of Red Cross/Crescent, Unicef, and the WHO) manage the allocation of OCV doses for outbreak response during emergency situations or humanitarian crisis. The Global Task Force on Cholera Control, a WHO coordinated network of partners, manages the allocation of OCV doses for vaccination in cholera endemic hotspots [36].

## 4. Dose-Sparing Approaches

Most of the OCV doses produced since 2013 enter the stockpile, which has increased from about two million doses per year in 2013–2014 to more than 17 million doses in 2018 [31]. Despite this increase, the availability of OCV doses remains limited compared with the population in need. Innovative OCV dose-sparing approaches have been evaluated.

### 4.1. Single-Dose Strategy

**Figure 1:**
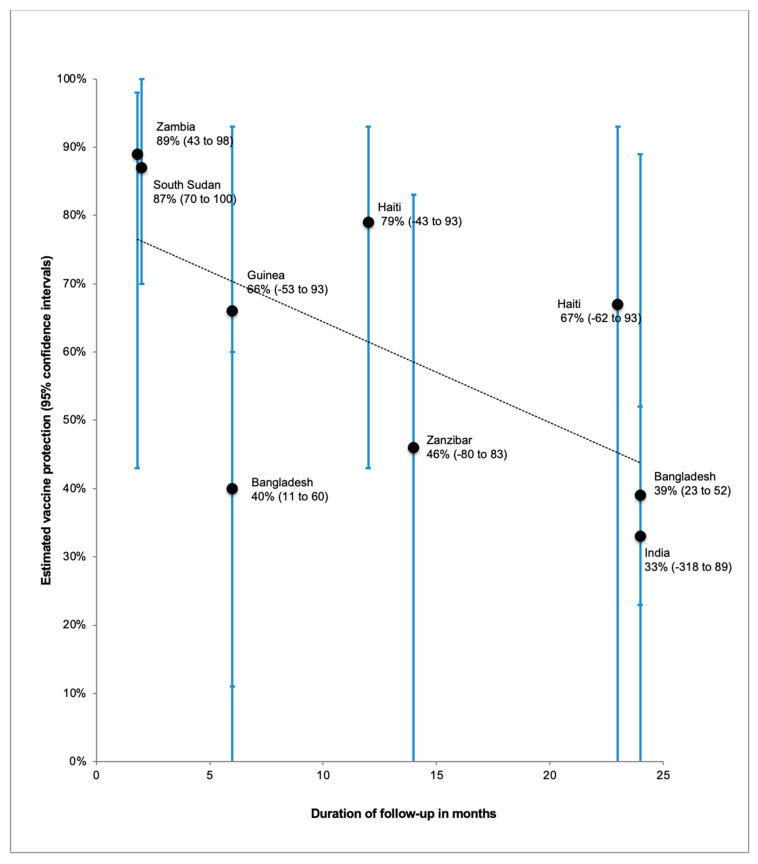
Estimated single-dose oral cholera vaccine protection (95% confidence intervals) with trendline, by month of follow-up. (Modified and updated from Lopez, A.L.; Deen, J.; Azman, A.S.; Luquero, F.J.; Kanungo, S.; Dutta, S.; von Seidlein, L.; Sack, D.A. Immunogenicity and Protection From a Single Dose of Internationally Available Killed Oral Cholera Vaccine: A Systematic Review and Meta-analysis. *Clinical Infectious Diseases*: 2018, 66, 1960–1971, doi:10.1093/cid/cix1039.)

A single dose regimen could mitigate against insufficient supplies and would also address the difficulties associated with delivery of two doses particularly during humanitarian emergencies, including accessing the same population twice, maintaining vaccine storage and retaining vaccination staff during the inter-dose period. A modeling study showed that reactive vaccination campaigns using a single dose of OCV may prevent more cases and deaths than a two-dose campaign when vaccine supplies are limited, while at the same time reducing logistical complexity [37]. Field evidence on OCV single-dose protection is available from one randomized controlled trial in Bangladesh [38,39] and several observational studies [24,27,40,41,42,43,44]. The protection conferred by a single dose was shown to be 89% at 7 weeks [43], waning to 39% at 2 years of follow-up [39] (Figure 1). Estimates of single-dose protection were generally lower in the randomized controlled trial than in the observational studies. Importantly, a subgroup analysis of the Bangladesh single-dose randomized trial found no significant protection in children younger than five years of age [38,39], which has been attributed to the lower pre-existing natural immunity in this age group.

Although the level of protection from a single OCV dose two years following vaccination is lower than the two-dose efficacy of 67% during the Kolkata trial [12], this may be sufficient to reduce the immediate short-term risk during outbreaks or in high-risk settings. A one-dose campaign, where more people receive a dose may be better in some circumstances than a two-dose strategy, where half as many people are vaccinated. In emergency situations, short-term protection is most critical and most of the public health benefit of reactive vaccination campaigns likely comes from the first dose, regardless of whether or not the second dose is administered [37]. However, the finding from the Bangladesh trial of no protective efficacy in young children suggests that the single-dose strategy may be beneficial only in populations with pre-existing natural immunity. Ideally, a second dose should be given as soon as circumstances allow to ensure longer and more robust protection, but this may not be possible due to inadequate OCV supplies or field logistics. 

In 2016, during a resurgence of cholera cases after Hurricane Matthew, Haiti launched a large emergency campaign when more than 700,000 people received a single dose of OCV [45]. During mass oral cholera vaccinations of Rohingya refugees in Bangladesh when only 900,000 doses were available, one dose was given to more than 700,000 people in October 2017, while a second dose was given in November 2017 to children between the ages of one to four years [46]. 

### 4.2. Targeted Deployment of OCVs

Another dose-sparing approach is the targeted deployment of OCVs, both as a pre-emptive or reactive strategy. Targeting discrete areas within a larger population at risk for cholera is usually necessary since the number of doses approved for allocation from the global stockpile is often less than the number requested. Criteria for the selection of targeted areas include the population size in an area in relation to the number of doses available, logistics required, historical attack rates of cholera and recent reported cases of cholera [47]. The concept of “source drying” may also be used when considering where to deploy limited number of OCV doses. For example, the two-dose mass vaccination campaign in Guinea targeted the Boffa and Forecariah coastal and island populations, which are highly mobile, have limited access to health care, safe water and basic sanitation and from whom cholera cases are often first reported during an outbreak [23,24].

In 2014, a two-dose OCV campaign was successfully conducted in selected areas of Kalemie, an urbanized and highly cholera-endemic area in the Democratic Republic of Congo [48] The targeted areas covered a population of around 120,000 people and had the highest historical attack rates in Kalemie. In 2015, a two-dose OCV campaign was carried out in ten selected villages of Shashemenae, a rural district of Ethiopia [49]. In 2015, 140,249 individuals in selected neighborhoods in Juba, South Sudan received a single dose of OCV in response to a cholera outbreak [50]. Targeting high-risk neighborhoods in Juba was done since authorities were unable to secure sufficient doses to vaccinate the entire at-risk population of about one million.

### 4.3. Ring Vaccination

When cholera outbreaks occur, there is usually broad agreement on the need for mass vaccination campaigns. In contrast, during smaller outbreaks or when sporadic cases occur in endemic areas, public health officials may be reluctant to allocate substantial resources for mass vaccination campaigns. Since cholera cases tend to cluster in time and place, particularly among household contacts of a cholera case [51,52], ring vaccination around cases could be considered. Ring vaccination may be used as a preliminary control strategy, which could be followed by a wider mass vaccination campaign if needed [53]. Data from the OCV efficacy trial in Kolkata [14] were used to model a potential OCV ring strategy and found that high-level protection can be achieved for those living close to cholera cases [54]. More recently, simulations of case-area targeted interventions, which can include improved water quality and supply, sanitation, hand washing, oral cholera vaccine, and prophylactic antibiotics, showed that vaccinating people within 100 m around index case households and improving their water source early in epidemics could reduce the number of cases by 82% compared to uncontrolled epidemics [55]. The addition of antibiotic treatment of neighbors within a 30-m to 45-m radius around the index case was helpful, but only in the short term. 

Ring vaccination using OCV may be less resource intensive than mass oral cholera vaccination but to be successful, cholera cases have to be detected quickly, sufficient OCV doses must be available on site within a short time from detection of the first cases, and the logistics for contact tracing and vaccination have to be set up immediately. A feasibility study in Nepal showed that cholera cases could be investigated within two days of a positive culture result [56]. The actual real-life feasibility and cost of integrating a sustainable cholera surveillance and ring OCV response system into a government’s health infrastructure has yet to be assessed. 

## 5. Evidence of Vaccine Herd Protection 

The term vaccine herd protection is widely used but carries a variety of meanings [57]. In this discussion, we define vaccine herd protection as the extension of the defense conferred by immunization beyond the vaccinated to unvaccinated persons in a population, as well as the enhancement of the protection among the vaccinated. Vaccine herd protection results from a decline in transmission of the pathogen within the community. Included in vaccine herd protection is the reduction in disease risk among the unvaccinated in the population (indirect protection) due to decreased exposure to the pathogen, as well as enhanced protection of vaccinees due to their proximity to other vaccinees (total protection). Unlike vaccinated individuals protected through direct immunity, individuals with indirect protection remain fully susceptible to infection, should they be exposed [57].

Aside from direct vaccine protective effects, there is increasing evidence of herd protection conferred by OCV. A reanalysis of a field trial in Matlab, Bangladesh demonstrated that OCV induces indirect protection of non-vaccinees, as well as enhanced protection of vaccinees [58]. A model of cholera transmission using information from the same trial showed that if about half the population was vaccinated, this would reduce the number of cholera cases among unvaccinated people by 89% and among the entire population by 93% [59]. For children too young to be vaccinated or to mount an adequate response to OCV (particularly to a single dose), based on principles of cocooning [60], oral cholera vaccination of older children and adults would be beneficial. There is evidence for substantial indirect protection of young children when a large proportion of older persons in the community are vaccinated [61]. 

More recently in Zanzibar, mass oral cholera vaccination was also found to confer indirect protection, as indicated by the lower risk of cholera in non-vaccinated individuals residing in areas with high vaccine coverage than in those residing in areas with low vaccine coverage [41]. Population-level effects of OCV was inferred from a study during the cholera epidemic in South Sudan in 2014 [62] The daily cholera reproductive number among internally displaced persons living in settlements that had received OCV vaccination was <1 for most of the epidemic, compared to >1 in unvaccinated areas even though conditions were less suitable for transmission in these unvaccinated areas.

The degree of population level effectiveness induced by a vaccine is driven by several factors, including vaccine-induced direct protection, vaccine coverage and population mixing and mobility [63]. A mathematical model of a simulated displaced-persons camp indicated that the duration of OCV-derived herd protection can be short in settings with high population mobility [64].

## 6. New Delivery Strategies

Mass oral cholera vaccination campaigns have generally utilized fixed posts for distribution [28], but other deployment methods may be used for various reasons. In the 2014 OCV campaign in Kalemie (described above), the vaccinations were administered door-to-door as it was feared that the targeted approach would generate tensions in the area, especially among those not selected for vaccination [48]. In October 2016, a two-dose pre-emptive mass vaccination campaign was given door-to-door in Nampula, Mozambique, which targeted 193,403 people [65]. The door-to-door method was used since this is the routine local distribution strategy for polio vaccination campaigns. 

OCV has been recommended to be stored at 2–8 °C but a study in Bangladesh showed that Shanchol has a good safety and immunogenic profile when stored under ambient temperature or even as high as 42 °C for up to 14 days [66]. Using OCV out of strict cold chain allows various possibilities for vaccine delivery and distribution. During the Guinea mass vaccination campaign, OCV doses were stored in cold chain but transported and used at ambient temperature during the vaccination days [23,24]. During a reactive two-dose OCV campaign in Lake Chilwa, Malawi, innovative strategies for the second vaccine dose (delivery by a community leader and self-administration) were used to facilitate vaccine access in hard-to-reach communities [67]. In another study in Dhaka, Bangladesh 41,694 people received a first OCV dose from fixed sites and the second dose was provided in a plastic zip-lock bag for the participant to take two weeks later at home [68]. Compliance for the second dose was estimated at 93% [68]. 

Cholera can cause serious complications in pregnant women and their fetuses if the disease is not treated promptly. Safety of the OCV during pregnancy has been demonstrated in several studies [69,70,71,72,73]. Pregnant women are no longer excluded during OCV mass campaigns [74].

## 7. Discussion

Since the availability of an effective OCV vaccine stockpile, more countries are open to acknowledging outbreaks and requesting OCV doses. The demand for OCVs will likely continue to outstrip supply in the near future. The constraints in supplies, complex logistics of administering the vaccine under difficult conditions and ensuring coverage of high-risk groups have resulted in alternative vaccination strategies, including single-dose regimens, targeted campaigns and locally adapted ways in administering OCVs. More recent campaigns have utilized a combination of these strategies. Although there is growing experience with the feasibility and acceptability of these methods, there is a need to continue documenting the protective effectiveness of OCVs when deployed using these methods.

In October 2017, the Global Task Force on Cholera Control launched an initiative to reduce cholera deaths by 90% worldwide, and eliminate cholera in at least 20 countries by 2030 [75]. A Global Roadmap to 2030 outlines three main axes for cholera prevention and control: early detection and rapid response to contain outbreaks; a multisectoral approach to prevent cholera in endemic countries (strengthening of surveillance, health care systems, water, sanitation and hygiene, and community mobilization and mass vaccination campaigns for communities at risk), targeting hotspots; and effective technical support, resource mobilization and partnership at local and international levels [76]. OCVs will play an important role to reach this ambitious goal but long-term improvements in WASH should be the ultimate aim.

## Figures and Tables

**Table 1:** Internationally Licensed and Recommended Inactivated Oral Cholera Vaccines [28,35].

Vaccine	Dukoral	Shanchol	Euvichol
Manufacturer	Valneva, Lyon, France	Shantha Biotechnics, Andhra Pradesh, India	Eubiologics, Gangwon-do, South Korea
Description	Monovalent inactivated vaccine	Bivalent inactivated vaccine	Bivalent inactivated vaccine
Components	Killed whole-cells of *V. cholerae* O1 (Classical and El Tor biotypes) and recombinant B-subunit of cholera toxin	Killed whole cells of *V. cholerae* O1 (Classical and El Tor biotypes) and *V. cholerae* O139	Killed whole cells of *V. cholerae O1* (Classical and El Tor biotypes) and *V. cholerae* O139
Recommended age	2 years and older	1 year and older	1 year and older
Delivery	Oral	Oral	Oral
Doses	2 doses given 1–6 weeks apart 3 doses for children aged 2–5 years	2 doses given 14 days apart	2 doses given 14 days apart
Buffer solution	Buffer dissolved in 75 mL (2–6 years old) or 150 mL (>6 years old) water	Not required	Not required
